# Comparison of the Antioxidant Activity of Propolis Samples from Different Geographical Regions

**DOI:** 10.3390/plants11091203

**Published:** 2022-04-29

**Authors:** Anna Kurek-Górecka, Şaban Keskin, Otilia Bobis, Rafael Felitti, Michał Górecki, Michał Otręba, Jerzy Stojko, Paweł Olczyk, Sevgi Kolayli, Anna Rzepecka-Stojko

**Affiliations:** 1 Department of Community Pharmacy, Faculty of Pharmaceutical Sciences in Sosnowiec, Medical University of Silesia in Katowice, St Kasztanowa 3, 41-200 Sosnowiec, Poland; polczyk@sum.edu.pl; 2 Vocational School of Health Services, Bilecik Seyh Edebali University, 11106 Bilecik, Turkey; sabankeskin61@hotmail.com; 3 Life Science Institute, Apiculture and Sericulture Department, University of Agricultural Sciences and Veterinary Medicine Cluj-Napoca, 400372 Cluj-Napoca, Romania; 4 Oral Rehabilitation and Prosthodontics, Private Practice, Felix Olmedo 3716, Montevideo 11700, Uruguay; rafael.felitti@gmail.com; 5 Department of Drug Technology, Faculty of Pharmaceutical Sciences in Sosnowiec, Medical University of Silesia in Katowice, St Jedności 8, 41-200 Sosnowiec, Poland; mgorecki@sum.edu.pl (M.G.); motreba@sum.edu.pl (M.O.); annastojko@sum.edu.pl (A.R.-S.); 6 Department of Toxycology and Bioanalysis, Faculty of Pharmaceutical Sciences in Sosnowiec, Medical University of Silesia in Katowice, St Ostrogórska 30, 41-200 Sosnowiec, Poland; jstojko@sum.edu.pl; 7 Department of Chemistry, Faculty of Science, Karadeniz Technical University, 61100 Trabzon, Turkey; skolayli61@yahoo.com

**Keywords:** antioxidant activity, phenolic acids, flavonoids, propolis

## Abstract

Propolis composition depends on several factors. The classification of propolis is based on its geographical location, color and agricultural characteristics. It is also classified according to the flora where the bees collect the resins, which represent the raw material for propolis production. Propolis possesses high antioxidant activity determined by its phenolic compounds. Due to diverse composition and possible impact on human health, eight samples of propolis were evaluated for their phenolic composition and antioxidant activity. Samples of Polish, Romanian, Turkish and Uruguayan origin propolis were used for phenolic spectrum determination using high performance liquid chromatography and photodiode array detection and in vitro DPPH and ABTS methods were used to determine the antioxidant activity of the extracts. PCA and HCA models were applied to evaluate the correlation between isolated polyphenols and antioxidant activity. The results confirmed variability in propolis composition depending on the geographical region of collection and the plant sources, and correlation between chemical composition and antioxidant activity. Results of PCA and HCA analyses confirm that Polish propolis is similar to that from different provinces of Romania, while Turkish and Uruguay are completely different. Polish and Romanian propolis belong to the poplar type. The assessed phenolic compounds of propolis samples used in the study are responsible for its antioxidant effect. The observed antioxidant activity of the analyzed samples may suggest directing subsequent research on prophylactic and therapeutic properties concerning cardiovascular, metabolic, neurodegenerative, and cancerous diseases, which are worth continuing.

## 1. Introduction

Propolis is a resinous substance collected by bees from plant buds, plant exudates, or resins found in the stem, branches, as well as leaves of different plants. Overall, raw propolis is composed of approximately 50% resins, 30% waxes, 10% essential oils, 5% pollens, 5% other organic substances [1]. Based on morphology, behavior and biological geography there are three major types of propolis: temperate region propolis; tropical region propolis; Pacific region propolis. Among the characteristic components of the first type of propolis are flavonoids without B-ring substituents, such as chrysin, galangin, pinocembrin, pinobanksin. The major component of temperate propolis possessing huge biological activity is caffeic acid phenethyl ester (CAPE), regardless of geographical origin [2,3]. The second type of propolis, characteristic for a tropical region, contains prenylated phenylpropanoids and diterpenes. The third type of propolis contains geranyl flavanones and is typically from the Pacific region or the African region [1]. The chemical categories of propolis should be considered according to the plant sources. The most common and world widespread propolis is the poplar type, derived from Europe, North America, non-tropical regions of Asia, New Zealand and even Africa. *Populus* species are the main plant source of propolis all over the world, especially in the temperate regions. The poplar type exhibits a typical chemical profile involving a high level of flavanones, flavones, low phenolic and their esters [2,3,4,5]. In the tropical and subtropical regions propolis is collected from poplar trees, but in the southeast of Brazil bees collect propolis from *Baccharis dracunculifolia*. The typical component of propolis from this botanical source is artepillin C. Moreover, propolis originated from Venezuela, Amazon, as well as Cuba, is collected from *Clusia* flowers and its chemical profile is characterized by prenylated benzophenones. The main botanical source of the next type of propolis— Macaranga - type Pacific propolis, occurring in Taiwan, and Okinawa, are the *Macaranga spp.* [6]. Moreover, Mediterranean propolis from Sicilia, Creta and Malta are collected from *Cupressus* plants. *Dalbergia* as a plant source is typically for red Brazilian propolis and Nepalese propolis [7,8]. *Eucalyptus* species are the main plant source in Australia, south Anatolia in Turkey, Egypt and Brazil. Due to the large diversity of plant sources of propolis, there is a need of further research, analysing chemical composition and antioxidant activities responsible for biological activities of propolis of different botanic origin [9,10].

Romanian propolis, as a poplar type propolis, have as main botanical origin resins from *Populus* species, but according to geographic regions of collection, also *Quercus, Aesculus, Ulmus, Picea, Salix* and *Fraxinus* have been reported as resin sources. Different studies demonstrate that Romanian propolis extract contains an average of 250–300 mg/g polyphenolic compounds, with a large variability according to the geographic region (landforms) [10,11,12], harvest time (month) [13], beekeeping practices (scraping from frames or propolis collector) [14] and last but not least extraction method [15,16]. Caffeic acid, chrysin, CAPE, and pinocembrin, being most commonly found, are accompanied by ferulic and *p*-coumaric acids, galangin, kaempferol and quercetin. Apigenin, rutin, luteolin, naringenin, and pinostrobin are also identified in propolis from different Romanian regions [10,11,12].

The Turkish propolis sample came from Anatolia. Propolis samples obtained throughout the Anatolia differ from each other in many ways depending on the floral sources. Anatolia can be divided into three phytogeographical regions. Plant sources available for propolis collection in these regions differ from each other and this situation is stated as a main reason for the difference between Anatolian propolis samples. According to the Turkish study, propolis was classified in three subtypes in terms of detailed chemical constituents, namely Orange type, Blue type and Third type. O-subtype was also mentioned to have maximum total phenolic content [17]. Many studies on the composition of Turkish propolis revealed that Turkish propolis are rich in phenolic acids like caffeic, ferulic, cinnamic acid and *p*-coumaric acid, as well as in derivatives and flavonoids, such as chrysin, galangin, pinocembrin and CAPE [17,18,19]. 

Polish propolis belongs to the poplar type. It is collected by the bees mainly from the leaf buds of the black poplar (*Populus nigra*) [20]. However, it can also come from birch (*Betula)* and alder (*Alnus*) [21,22]. According to the Polish researchers, the characteristic propolis compounds are polyphenols. The amounts of total polyphenols in Polish propolis is about 58%, while in the condensed extract, it is about 78% [23]. The main components of Polish propolis, responsible for its biological properties, are phenolic compounds including flavonoids, phenolic acids and their esters. Polish propolis is rich in flavonoids such as chrysin, apigenin, galangin, kaempferol, pinostrobin, pinobanksin, naringin, quercetin and phenolic acids like caffeic, ferulic, gallic, benzoic, cinnamic, coumaric and vanillin acid as well as their esters [22]. Among the most valuable aromatic esters from Polish propolis, there are benzoic acid benzyl ester, cinnamic and caffeic acid ether and benzoic acid phenylmethyl ester [24].

Uruguayan propolis contains compounds from different classes of substances, such as flavonoids, four aromatic carboxylic acids and eleven phenolic acid esters. Of these compounds, should be enumerated pinobaskin and its derivates, i.e., pinobanksin 3-hexaonate, pinobanksin 3-butanoate, pinobanksin 3-propanoate, pinobanksin 3 acetate, pinobanksin 3-acetoxy-7-methyl ester and pinobanksin, pinostrobin and pinocembrin. The kind of propolis mentioned also contains other flavonoidic compounds like chrysin, tectochrysin, galangin, kaempferol, and moreover, aromatic carboxylic acids like *p*-coumaric and caffeic acid, as well as aromatic carboxy acids esters [25,26].

The aim of this study was to evaluate the antioxidant properties of different types of propolis from various geographical regions. The chemical composition of propolis is responsible for its antioxidant effect. In our study, the antioxidant activity in vitro of eight propolis samples from the four mentioned countries, i.e., Romania, Turkey, Poland and Uruguay have been evaluated.

## 2. Results and Discussion

This study demonstrates the antioxidant activity of eight samples of propolis originating from different geographical regions. In order to determine the antioxidant activity of propolis samples, ethanolic extracts of each sample of propolis were obtained with two step extractions. Finally, extracts were established at a concentration of 1 mg/mL. The research of antioxidant activity was based on electron donation mechanism as DPPH assay, and partly on ABTS that are stabilized after hydrogen donation. Results of the antioxidant activity assays are correlated to polyphenolic profile of propolis samples. The polyphenolic profile of each sample was determined by HPLC. The results of this in vitro assay show the relation among phenolic content (total phenolic content, total flavonoids and individual phenolics), plant origin and antioxidant properties.

### 2.1. DPPH Radical Scavenging Activity

**Figure 1:**
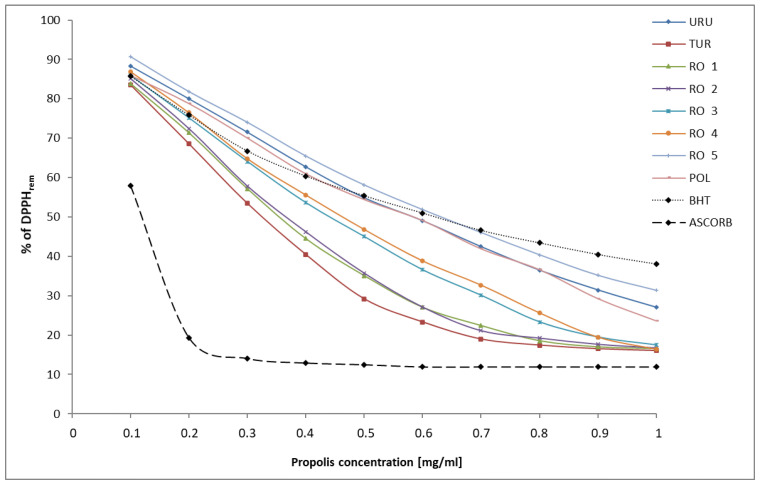
Percentage of remaining DPPH radical at different propolis samples (URU—Uruguayan, TUR—Turkish, POL—Polish, RO 1–5—Romanian) and known antioxidants such as: butylhydroxytoluene (BHT) and ascorbic acid (ASCORB). The standard deviation (SD) of the three independent determinations of each concentration of each propolis samples has not exceeded the values as follows: URU ± 4.7, TUR ± 3.0, POL ± 3.4, RO1 ± 7.0, RO2 ± 3.1, RO3 ± 5.6, RO4 ± 4.8, RO5 ± 4.4. Points on graphs represent the average.

DPPH constitutes a stable organic free radical, which loses its absorption band at 517 nm by accepting an electron or a free radical species. This assay is a popular method being used to evaluate an antioxidant activity of natural products including propolis. The ability of propolis to reduce DPPH was measured and EC_50_ values were defined as amounts of ethanol extract of propolis giving 50% DPPH reduction. Moreover, it may be expressed as remaining DPPH, which means the amount of unreduced DPPH radical. The percentage of remaining DPPH radical in the analyzed propolis samples is presented in Figure 1.

In this study, the highest DPPH radical scavenging activity with minimum EC_50_ was observed for Turkish propolis (EC_50_ = 0.325 mg/mL), followed by Romanian propolis 1 (EC_50_ = 0.355 mg/mL), Romanian propolis 2 (EC_50_ = 0.365 mg/mL), Romanian propolis 3 (EC_50_ = 0.440 mg/mL), Romanian propolis 4 (EC_50_ = 0.460 mg/mL). Polish propolis and Uruguay propolis exhibited the same value EC_50_ equal 0.585 mg/mL. The lowest DPPH radical scavenging activity has been demonstrated in Romanian propolis 5. Its value of EC_50_ was estimated as 0.630 mg/mL. All results were compared with the EC_50_ value of two standards: BHT—butylhydroxytoluene (EC_50_ = 0.365 mg/mL) and ascorbic acid (EC_50_ = 0.103 mg/mL). Therefore, the antioxidant activity of Turkish and Romanian (RO1) propolis was better than BHT. Romanian propolis (RO2) presented a similar activity compared to BHT. However, ascorbic acid exhibits three–six times higher antioxidant activity, compared to that of propolis from different countries.

### 2.2. ABTS Radical Scavenging Assay

**Figure 2:**
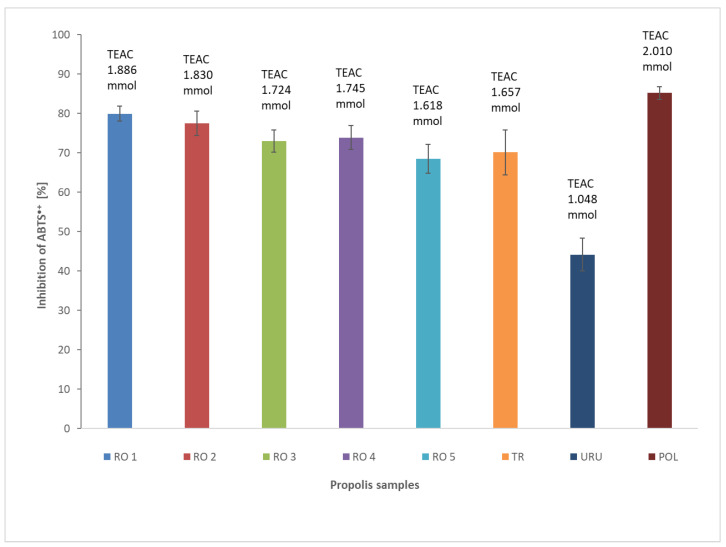
The percentage of ABTS radical cation inhibition at different propolis samples and calculated TEAC values (URU—Uruguayan, TUR—Turkish, POL—Polish, RO 1–5—Romanian). Error bars represent the standard deviation (SD) of the three independent determinations.

Another in vitro method that allows us to estimate the antioxidant activity of propolis is a method using ABTS radical cation to measure the relative ability of propolis to scavenge the ABTS as compared with a Trolox (water soluble vitamin E analogue) standard (Figure 2). This method can be used to evaluate both hydrophilic and lipophilic antioxidants [27]. Based on spectrophotometric measurements, the values of the percentage of inhibition and TEAC for propolis extract at a concentration of 1 mg/mL were calculated. The higher the percentage of inhibition, the greater the antioxidant propolis properties. The percentage of inhibition (PI) [%] in reduced ABTS radical cation for different propolis samples is presented in Figure 2. 

TEAC determines the amount of Trolox equivalent per unit of sample volume. A higher TEAC value is related to the greater antioxidant properties. TEAC value of each propolis sample has been calculated based on a calibration curve for Trolox y = 0.2828x – 0.0035 R^2^ = 0.9998 (Figure 2). The highest ABTS radical scavenging activity was exhibited in the propolis sample from Poland. The measurement of TEAC for Polish propolis (POL) was equal to 2.01 mmol. Samples of propolis that came from Romania showed a high value of TEAC as follows: 1.886 mmol for sample 1 (RO1), 1.830 mmol for sample 2 (RO2), 1.745 mmol for sample 4 (RO4), and 1.724 mmol for sample 3 (RO3). The value of TEAC for Turkish propolis (TUR) was 1.657 mmol. A lower value than Turkish propolis was found in the case of propolis RO5—1.618 mmol. The lowest antioxidant activity displayed was Uruguayan propolis (URU), revealing a TEAC value equal to 1.048 mmol. Both in estimating an antioxidant activity with DPPH and ABTS, a propolis sample from Uruguay exhibited the lowest antioxidant activity. The observed differences in results concerning a Romanian propolis indicate that not only geographical region, but also the chemical composition of propolis derived from a diverse botanical source, have an impact on antioxidant properties. The antioxidant activity of the investigated samples was higher, compared to propolis from Lithuania, although total phenolic content and total flavonoids are similar in quantity [5]. Accordingly, it can be pointed out that phenolics—a type of active compounds that are a major determinant of the antioxidant potential of propolis, which was confirmed by Olczyk et al. [28].

Therefore, the determination of phenolic composition of each propolis sample was necessary. Among analyzed samples, there were selected phenolic compounds. We prepared eight ethanol extracts from propolis samples, each was purified and quantified by HPLC to provide a total: thirteen phenolic compounds, involving phenolic acids, such as gallic acid, caffeic, *p*-coumaric acid, ferulic and *t*-cinnamic acid, as well as flavonoids such as luteolin, quercetin, apigenin, hesperidin, rhamnetin, chrysin, pinocembrin and one aromatic ester, i.e., CAPE, were identified by HPLC and measured quantitatively.

According to Kumazawa et al. [25] Uruguayan propolis has a plant origin similar to that of propolis from Europe and China, therefore it probably contains similar compounds to the latter one [22]. Thus, the further studies on a higher number of samples are needed to identify the correlation among the type of phenolic compounds, their amount and the interaction between them, to explain the lower antioxidant activity of this propolis type, i.e., Uruguayan propolis.

### 2.3. Bioactive Compounds and Correlation between Propolis Content of Phenolic Compounds and Antioxidant Activity

The content of total polyphenols and flavonoids from the extracts has been evaluated spectrophotometrically. Total phenolic content and flavonoids per g of dry extract of propolis samples from different countries is presented in Table 1. 

The in vitro antioxidant activity was analyzed using DPPH and ABTS methods. Based on the results of antioxidant activity assessed with radical DPPH, there is a strong negative correlation between the content of polyphenols and EC_50_ value of DPPH concerning different samples of propolis. The value of Spearman correlation coefficient is −0.8503. The lowest value of EC_50_ indicates the highest antioxidant activity of the tested samples. As can be seen from the obtained results, a classical analysis of matrix (spearman’s correlation) revealed a little difference between the compared variables. There is no significant correlation between the total amount of polyphenols and ABTS assay. Similarly, there is no correlation between the total amount of flavonoids and antioxidant activity measured by DPPH and ABTS methods. In order to assess the connection between isolated polyphenols and antioxidant activity, multivariate analysis is proper. Therefore, in this case a principal component analysis (PCA), and hierarchical clustering analysis (HCA) should be applied. What is more, an identification of phenolic compounds from extracts will allow us to explain their impact on antioxidant propolis activity.

Polyphenols are plant secondary metabolites and their biosynthesis is mediated by different enzymes, specific for different plant species, plant needs and oxidative stress. The plant origin of the resin collected by the bees determines the type of phenolics, which are present in propolis from different countries. Therefore, the chemical composition of different propolis extracts was evaluated by high performance liquid chromatography. The detected phenolic compounds in the analyzed samples are presented in Table 2. Among the studied propolis samples, five phenolic acids such as gallic, caffeic, *p*-coumaric, ferulic and *t*-cinnamic acid have been identified. All studied samples contained the above-mentioned phenolic acids. Extracts were made in triplicate. For the HPLC analysis, a sample representing a pull of the extracts was analyzed and results are presented in the following table. 

The highest amount of gallic acid was identified in the Polish sample. In all Romanian samples, gallic acid was also identified, in higher amounts than in the Turkish and Uruguayan samples. Caffeic and *p*-coumaric acid were also identified in all analyzed samples. Sample 2 from the Romanian propolis presented the highest amount of caffeic acid (778.371 μg/g), followed by the Turkish sample, RO1, RO4 and POL. The Polish and RO2 samples presented the highest amounts of *p*-coumaric acid (3452.608–3547.561 μg/g). Other Romanian samples were also rich in *p*-coumaric acid, although the highest amount of this compound has been quantified in RO2. The Turkish and Uruguayan propolis extracts presented very low amounts of *p*-coumaric acid (455.273–277.124 μg/g). Ferulic acid was present in the highest amount in sample 2 from the Romanian propolis (1114.551 μg/g), followed by RO1, RO4 and the Turkish propolis samples. The highest amount of *t*-cinnamic acid was determined in the Turkish propolis sample (235.758 μg/g), followed by POL, RO3, URU, RO2, RO4, RO1, RO5 samples, in which much smaller amounts (23.354–69.213 μg/g) of the mentioned compound were found. Only the propolis sample from Turkey displayed a luteolin and rhamnetin content. Regarding quercetin, it was present in samples from Poland, Turkey and three Romanian samples (RO3, RO4 and RO5). Apigenin was present in samples from Poland, Turkey and Uruguay (124.953–219.721 μg/g). Hesperidin was present in high amounts in Uruguayan (138.938 μg/g), and in Turkish propolis (69.263 μg/g). In all other samples this flavonoid was not identified. Chrysin, a propolis characteristic compound, was identified and quantified in all investigated samples in different amounts. The highest amounts were determined in Turkish and Uruguayan propolis (>2000 μg/g), then in Polish and RO3, RO4 (>1000 μg/g), RO1, RO2 (>500 μg/g) samples, respectively. Pinocembrin was also quantified in the highest amounts in propolis samples from Turkey and Uruguay (1726.058–2816.289 μg/g), followed by RO3 samples (916.312 μg/g). The differences between the quantities of phenolic compounds in analyzed extracts were statistically significant (*p* < 0.05). In all samples, caffeic acid phenethyl ester (CAPE) was identified. The last one is a major propolis active compound and is thought to be responsible for many of its biological activities, such as being antibacterial, antiviral, antifungal, antioxidant, anti-inflammatory and anticancer [12,29].

The highest content of CAPE was quantified in propolis from Turkey (1118.623 μg/g). The highest radical scavenging activity of Turkish propolis observed in assay with a radical DPPH may be a result showing a high content of this compound with strong antioxidant activity. According to Sulaiman et al., CAPE possesses better radical scavenging activity compared to chrysin as a result of better electron delocalization in radical form [30].

Different chromatographic techniques (high performance liquid chromatography tandem mass spectrometry and NMR spectroscopy; gas-chromatography coupled with mass spectrometry) are used for a more precise characterization of propolis samples all over the world [31,32]. Further studies are needed for the studied samples, to confirm the difference between samples from different geographic origins in their individual phenolic profile and possible marker determination. 

Literature data indicate that antioxidant activity is associated with the total content of phenolic compounds. This fact has also been confirmed in the present study. Propolis from Turkey was found to contain the highest amount of *t*-cinnamic acid, quercetin, chrysin and pinocembrin of the all analyzed samples. Moreover, it is rich in ferulic acid and apigenin. These compounds influence its strong antioxidant activity. The antioxidant assays, ABTS radical cation assay and DPPH assay, confirmed that propolis samples containing the highest amount of phenolic compounds exhibit the highest antioxidant capacity. According to the assay with ABTS, a radical cation inhibition strong activity has been demonstrated by propolis from Poland. The last phenomenon may be connected with the highest content of phenolic acids such as gallic acid and *p*-coumaric acid. Moreover, apigenin was found to occur in highest amount in the Polish propolis sample. The structure of propolis polyphenols may explain this observation, which has been confirmed by Okińczyk et al. [33]. It is known, that the presence of additional hydroxyl groups in the aromatic polyphenol ring creates the antioxidant propolis activity. Moreover, the substitution of the hydrogen atom in the hydroxyl group with a methyl group influences the antioxidant activity in a different way, depending on the polarity of the environment in which the antioxidant activity is studied [34]. Benzoic acid dihydroxy derivatives display the most powerful anti-oxidative properties when the hydroxyl groups appear in the positions three and five. Among benzoic acid derivatives, gallic acid, containing three hydroxyl groups in the three, four and five positions, exhibits high antioxidant activity [23]. However, the derivatives of cinnamic acid better demonstrate anti-oxidative properties than benzoic acid derivatives. In the case of an ethylene group introduced between the phenyl ring containing a hydroxyl group in the para-position and a carboxyl group (e.g., *p*-coumaric acid), the increase in the reductive properties of the hydroxyl group, in comparison with cinnamic acid, is observed. Apart from phenolic acids, flavonoids influence an antioxidant activity. According to recent literature data, apigenin (4′,5,7-trihydroxyflavone) exhibits antioxidant and anticancer properties [35]. These features were confirmed by the ABTS radical cation assays, which revealed a correlation between an antioxidant activity and the presence of different flavonoids in apigenin molecules.

### 2.4. Multivariate Statistical Interpretation

**Figure 3:**
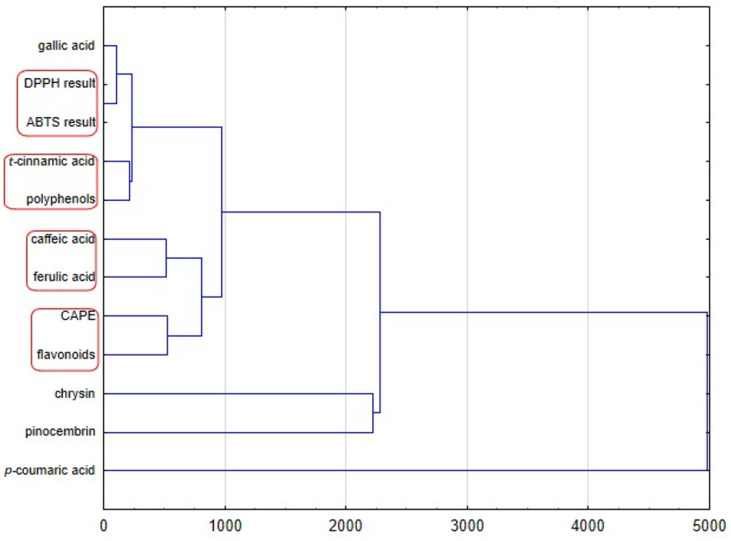
HCA dendrogram obtained by analysis of bioactive compounds identified in tested propolis samples.

**Figure 4:**
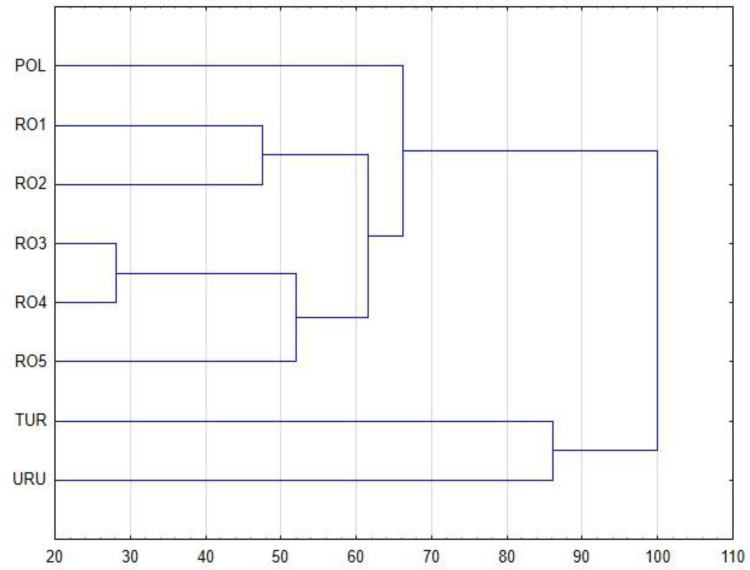
HCA dendrogram obtained by analysis of propolis of different origin.

**Figure 5:**
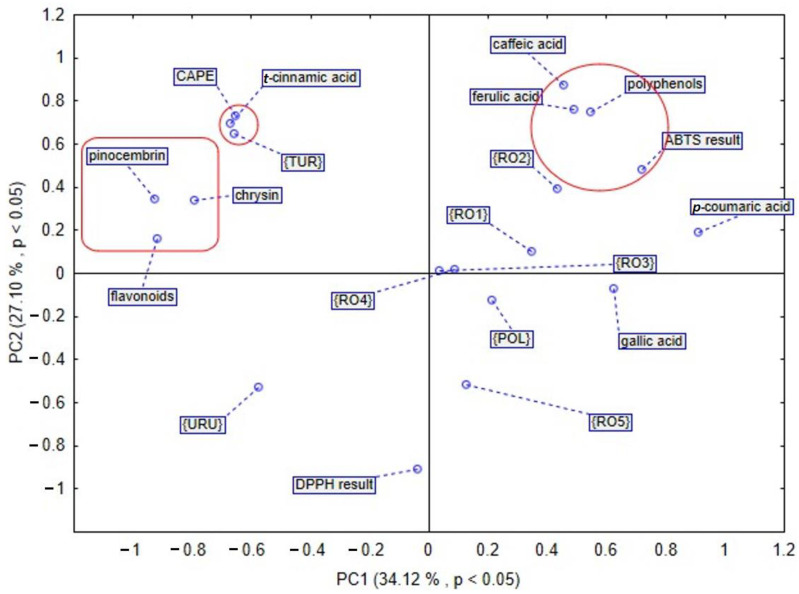
PCA score plot obtained by analysis of bioactive compounds identified in tested propolis samples. This PCA was based on analysis of detected bioactive compounds derived from different propolis samples.

**Figure 6:**
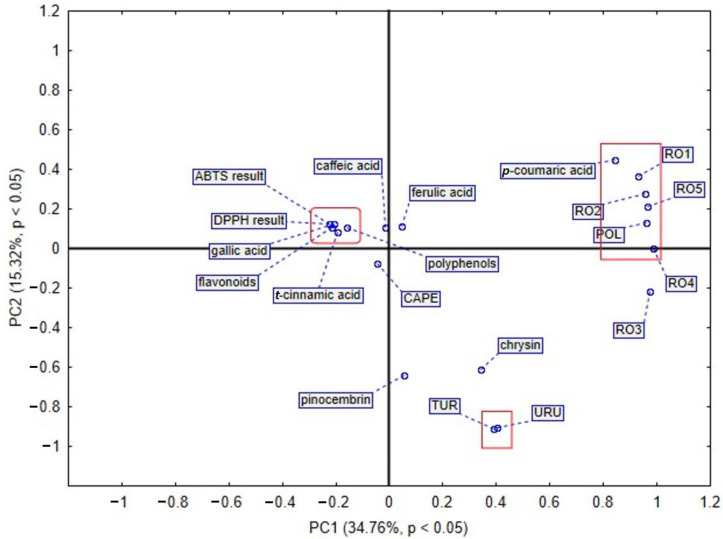
PCA score plot obtained by analysis of propolis of different origin. This PCA was based on analysis of different propolis samples analyzed in terms of the total amount of detected bioactive compounds.

Since the studied propolis samples are of variable botanical diverse composition, principal component analysis (PCA) and hierarchical clustering analysis (HCA) were conducted to sort the propolis samples. The obtained results analyzed by HCA are shown in Figure 3 and Figure 4, while results analyzed by PCA are shown in Figure 5 and Figure 6. In the case of PCA, the first two components explained 61.22% of the data variance, and PC1 explained 34.12% of the mentioned variance, whereas PC2 explained 27.10% (Figure 5). PCA brought satisfactory results and allowed us to differentiate the propolis due to some groupings of the samples being noticed in the PCA score plot.

According to the PCA presented in Figure 5, four main groups were identified. The first selected group includes Turkish propolis. It contains the highest amount of CAPE (1118.623 µg/g dry extract) and *t*-cinnamic acid (235.758 µg/g dry extract), respectively. Moreover, among flavonoids, a pinocembrin and chrysin were also detected in the largest amount in sample from Turkey. The second selected group shows that polyphenols have a huge impact on antioxidant activity. This phenomenon was revealed by the results of assay with ABTS radical cation. Among phenolic acids, caffeic and ferulic acid play an important role in the antioxidant potential of propolis. Samples of the mentioned natural product from Romania, mainly RO2 and RO1, contain a high amount of caffeic acid. Furthermore, the Romanian sample RO4 distinguished itself in the amount of that phenolic acid. Similarly, ferulic acid was detected in the highest amount in Romanian propolis, RO2 and RO1. The analysis of the third selected group demonstrates that the chemical composition of propolis from Uruguay is different from the others. The fourth selected group in the PCA plot indicates that Polish propolis is similar to that from Romania, taking into consideration bioactive compounds. What is more, gallic acid was found to be present in the largest amount in the Polish propolis, as compared to propolis samples of different origin. In another PCA based analysis of different propolis samples, we took into consideration the amount of bioactive compounds presented in Figure 6, and as a result, we observed that the first two components explained 50.08% of the data variance, PC1 explained 15.32% of the mentioned variance, whereas PC2 explained 34.76%. In this PCA sore plot three main groups were identified. The first selected group shows that polyphenols and flavonoids are connected with a propolis antioxidant activity assayed by DPPH and ABTS radical cation methods. The second selected group confirms that Polish propolis is similar to the Romanian one and contains a large amount of *p*-coumaric acid. Propolis from Romanian Province 2 contains the highest amount (3537.61 µg/g dry extract), whilst Polish propolis contains 3452.608 µg/g dry extract of *p*-coumaric acid. The third selected group includes Turkish and Uruguayan propolis. These samples are different from the others in terms of their components and antioxidant activity. 

The dendrogram of the chemical profiles of propolis samples obtained by HCA (complete linkage using Euclidean distances) is presented in Figure 3. This dendrogram presents four groups. The first cluster indicates that the antioxidant activity of different propolis samples measured by two methods with DPPH and ABTS is most similar, as this is the cluster with the lowest Euclidean distance value. The total amount of polyphenols is correlated with amount of *t*-cinnamic acid. The quantity of caffeic and ferulic acid influences the antioxidant properties, especially when these acids are present together. The last of our observation shows that the amount of flavonoids and CAPE have an impact on the antioxidant properties of analyzed propolis samples. Moreover, another HCA analysis (Figure 4) presents an Euclidean distance among propolis samples of different origin. It confirms the results of PCA analyses. Polish propolis is similar to that from different provinces of Romania, while Turkish, and Uruguayan, are completely different, which is probably at least partially due to different plants being a source of natural bee product-propolis.

## 3. Materials and Methods

### 3.1. Materials

Gallic acid (3,4,5-trihydroxybenzoic acid), Caffeic acid, *p*-Coumaric acid, Ferulic acid, Luteolin, Quercetin, *t*-Cinnamic acid, Apigenin, Hesperidin, Rhamnetin, Chrysin, Pinocembrin, Protocatechuic acid, Chlorogenic acid, *p*-hydroxybenzoic acid, Epicatechin, Syringic acid, *m*-hydroxybenzoic acid, Ellagic acid, Myricetin, Resveratrol, Daidzein, Curcumin, Caffeic acid phenethyl ester (CAPE), 2,2-diphenyl-1-picrylhydrazyl (DPPH), Ascorbic acid were obtained from Sigma-Aldrich. Butylated hydroxytoluene (BHT), 2,2′-azino-bis(3-ethylbenzthiazoline-6-sulfonic acid) (ABTS), 6-hydroxy-2,5,7,8-tetramethylchroman-2-carboxylic acid (Trolox), were purchased from Fluka, USA. Potassium persulfate, Sodium carbonate, Folin–Ciocalteu solution, Aluminium chloride, Ethanol 96%, Methanol, were purchased from POCH, Poland.

### 3.2. Samples of Propolis

One sample of propolis from Uruguay, Turkey, Poland and five samples of propolis from Romania, collected in different geographic areas and altitude zones with typical continental climate conditions and different vegetation were analyzed for the chemical composition and bioactive properties. Raw samples were frozen upon receiving and grinded before alcoholic extraction. All samples had a typical smell characteristic of resin, specific propolis colors (dark yellow to reddish brown). Names and origin of propolis samples used in this research are presented in Table 3.

### 3.3. Preparation of Ethanol Extract of Propolis

The following samples of raw propolis deriving from enumerated countries were obtained: one sample from Poland (POL), one sample from Uruguay (URU), one sample from Turkey (TUR) and five samples from Romania (RO 1–5). The samples were ground mechanically. Twenty grams of each propolis sample was extracted with 100 mL of 70% ethanol (*w*/*v*). For extraction, the flasks were placed in the dark for one week at room temperature. Obtained extracts were filtered and collected separately. The sediments after filtrations were re-extracted with 100 mL of 70% ethanol (*w*/*v*) in the same conditions. After filtration, the extracts were joined and cooled at 4 °C for 24 h to precipitate all insoluble particles. After a final filtration, extracts were evaporated to dryness at 40 °C using a rotary vacuum evaporator. The extraction efficiency (expressed as a percentage) was calculated as a weight ratio of dry mass of the extract obtained to the mass of raw propolis used. The efficiency of two-step extraction was as follows: POL 54%, URU 51%, TUR 55%, RO1 53%, RO2 53%, RO3 56%, RO4 52%, RO5 51%. To receive the final concentration of each extract solution at 1 mg/mL, 100 mg of dried extract was dissolved in volumetric flask in 70% ethanol (*w*/*v*) to a final volume of 100 mL.

### 3.4. DPPH Radical Scavenging Activity

The antioxidant activities in vitro of propolis ethanol extracts were determined using DPPH free radical [36]. In order to investigate a DPPH radical scavenging activity of propolis samples, a dilution series was prepared for each extract as follows: 0.1 mg/mL; 0.2 mg/mL; 0.3 mg/mL; 0.4 mg/mL; 0.5 mg/mL; 0.6 mg/mL; 0.7 mg/mL; 0.8 mg/mL; 0.9 mg/mL; 1 mg/mL. To each diluted solution of propolis extract (0.1 mL), 3.9 mL of methanol DPPH solution at concentration of 6 × 10^−5^ M/L was added. The decrease in measured absorbance was evaluated at 517 nm after 30 min. The blank sample consisted of 0.1 mL 70% ethanol and 3.9 mL of methanol DPPH solution. BHT solutions at concentrations of: 0.4 mg/mL; 0.3 mg/mL; 0.2 mg/mL; 0.15 mg/mL 0.1 mg/mL; 0.05 mg/mL and ascorbic acid at concentrations of: 0.2 mg/mL; 0.15 mg/mL; 0.1 mg/mL; 0.075 mg/mL; 0.05 mg/mL were used to prepare standard curves. Percentage of inhibition (PI) (%) was calculated as the percentage of reduced DPPH radical. It was calculated from the Formula (1):

(1) $$\mathrm{PI}=\left(\frac{A_{\mathrm{blank}}-{\;A}_{\mathrm{sample}}}{A_{\mathrm{blank}}}\right)\times 100\%,$$

where: A_sample_—absorbance of propolis sample; A_blank_—absorbance of blank sample.

DPPH_rem_ [%] was calculated from the Formula (2):

(2) $${\mathrm{DPPH}}_{\mathrm{rem}}=\left(\frac{A_{\mathrm{sample}}}{A_{\mathrm{blank}}}\right)\times 100\%,$$

where: A_sample_—absorbance of propolis sample; A_blank_—absorbance of blank sample.

The experiment was performed in triplicates. Based on the graphical chart presenting the dependence of % DPPH_rem_ on the concentration of propolis extract, the value EC_50_ (mg/mL) has been expressed as a concentration of propolis that caused a decrease of about 50% of the starting concentration of DPPH radical and calculated for all extracts.

### 3.5. ABTS Radical Scavenging Assay

The ABTS assay is based on the ability of antioxidants to scavenge the ABTS generated in the aqueous phase, as compared with Trolox (water soluble vitamin E analogue) standard. The ABTS is generated by reacting the ABTS salt with a strong oxidizing agent as potassium persulfate in ratio 1:0.5. The propolis samples have been prepared at concentration 1 mg/mL. Briefly, 0.04 mL of propolis solution (1 mg/mL) was added to 4 mL solution of ABTS^•+^ radical cation and the mixture was kept in the dark at room temperature. The decrease in measured absorbance was evaluated at λ = 734 nm after 30 min. The blank sample consisted of 0.04 mL 70% ethanol and 4 mL of ABTS^•+^ radical cation solution. Trolox solutions at concentrations: 1 mM; 0.75 mM; 0.5 mM; 0.25 mM and 0.1 mM were used to prepare a standard curve. The percentage of inhibition (PI) was calculated as the percentage of reduced ABTS radical cation from the Formula (1). The experiment was performed in triplicates.

### 3.6. Total Phenolic Content

The phenolic content of the propolis samples at a concentration of 0.2 mg/mL was assessed by the Folin–Ciocalteu method and expressed as a gallic acid equivalent [37].

1 mL of propolis ethanol extract, 1 mL Folin–Ciocalteu solution and 1 mL of 5% sodium carbonate solution (Na_2_CO_3_) were mixed. The mixture was incubated for 60 min in the dark at room temperature. The absorbance was evaluated at 760 nm. Total phenolic content was estimated from a standard curve of absorbance values derived from standard concentration solutions of gallic acid. Gallic acid in serial dilution (0 mg/mL; 0.1 mg/mL; 0.2 mg/mL; 0.3 mg/mL; 0.4 mg/mL and 0.5 mg/mL) was prepared as standard for constructing the calibration curve. Total phenolic content in the tested samples was determined from the linear regression equation of the standard curve (y = 16.583x – 0.074, R^2^ = 0.9946) and it has been expressed in gallic acid equivalents. The experiment has been performed in triplicates.

### 3.7. Total Content of Flavonoids

The flavonoids content of the propolis samples at a concentration 0.2 mg/mL was assessed by the method of Woisky and Salatino [38] and expressed as quercetin equivalent per g of dry extract. The aluminum chloride (AlCl_3_) was used as a reagent to determine the flavonoids content in the propolis ethanolic extracts. A total of 1 mL of ethanol extract of propolis and 1 mL 2% AlCl_3_ methanolic solution were mixed. The mixture was incubated for 60 min in the dark at room temperature. The absorbance was evaluated at 420 nm. Total flavonoids content was estimated from a standard curve of absorbance values derived from the standard concentration solution of quercetin. Quercetin in serial dilution (0.02 mg/mL; 0.025 mg/mL; 0.03 mg/mL; 0.035 mg/mL; 0.040 mg/mL; 0.045 mg/mL and 0.05 mg/mL) was prepared as a standard for constructing the calibration curve. The total content of flavonoids in the tested samples was determined from the linear regression equation of the standard curve (y = 32.543x + 0.0607, R^2^ = 0.9974).

### 3.8. HPLC Analysis

The phenolic composition of each sample was determined by using a modified method described by Can et al. [39]. Briefly, dried propolis extracts were handled to re-extraction for HPLC analysis by dissolving them in 10 mL acidified (pH 2) distilled water separately. The solution was acidified to pH 2 by adding hydrochloric acid. Re-extraction was carried out firstly with 5 mL diethyl ether, and then with 5 mL ethyl acetate, for three times each. Solvent phases were combined separately. Residues obtained after removing the solvents at 45 °C were re-dissolved in 2 mL of methanol and filtered with 0.45 µm filter. The filtrates were injected with HPLC. Twenty five phenolic standard compounds were analyzed using HPLC (Elite LaChrome; Hitachi, Tokyo, Japan) equipped with a reverse phase C18 column (150 mm × 4.6 mm, 5 µm; Fortis). Acetonitrile/water (7/3 ratio) and acetic acid 2% were used as mobile phase and a programmed gradient was applied. Reservoir A contained 2% acetic acid and reservoir B contained 7/3 ratio of acetonitrile/water. Then, 20 µL of the sample was injected individually at room temperature and a flow rate was set at 0.75 mL/min [40]. For quantitative determination, a calibration curve of each phenolic component was constructed with R^2^ ranging between 0.998 and 1.000. The detection of the chemicals was carried out by a UV–Vis detector, supplying a double wavelength at 280 and 315 nm, simultaneously [41]. Measurements were made in triplicate and results were expressed as mean value ± standard deviations.

### 3.9. Statistical Analysis

Statistical analysis of the data was performed with Statistica13.1 software (StatSoft Inc., Tulsa, OK, USA). HPLC results were evaluated using Kruskal–Wallis ANOVA. Results were expressed as the mean ± SD of three independent experiments, which were conducted, in triplicates. The level of statistical significance (*p*) values lower than 0.05 were considered significant.

The data obtained were also analyzed using hierarchical cluster analysis (full linkage using Euclidean distance) and principal component analysis (PCA). The PCA model (based on standardized data) was estimated using the NIPALS iterative algorithm. The criterion of convergence was set at the level of 0.00001 and the maximum number of iterations was set at 50. The number of components was assessed by determining the maximum predictive capability using the method of multiple cross-validations and the maximum number of components. The obtained optimal PCA model was then reduced to two components. The PCA analysis, the results of which are presented on the chart of PC 1 vs. PC 2 loads, allowed us to select variables having the most significant influence on the variability of the analyzed database of results, and to select the most significant correlations between them. The variables selected in this way were then subjected to further statistical evaluation. These two classification techniques (PCA and HCA) were used to discover natural groupings in the data and examine differences between the analyzed propolis ethanol extracts.

## 4. Conclusions

The study was carried out to evaluate the antioxidant activity in vitro of different compounds of propolis of distinct origin. The studies confirmed variability in propolis composition, depending on the geographical region of collection and the plant sources. Moreover, the correlation between chemical composition and antioxidant activity of the different propolis samples was found. The antioxidant assays, including ABTS assay and DPPH assay, confirmed that propolis samples rich in compounds with strong antioxidant activity exhibit the highest antioxidant properties. Moreover, the samples that came from Romania exhibited diversity, displaying a difference in terms of antioxidant activity of particular samples. Thus, the propolis extracts from Romania Province 1 (RO1) had the highest DPPH radical scavenging activity, while the lowest one was in the case of propolis from Romania Province 5 (RO5). Interestingly, when only EEP from Romania propolis samples were considered, strong differences among propolis harvested in different provinces were observed. The highest ABTS radical scavenging activity exhibited propolis from Romania Province 1 (RO1) and the lowest from Romania Province 5 (RO5).

Additionally, the antioxidant activity of propolis samples was found to be associated with a content of phenolic compounds. Propolis from Turkey turned out to contain the highest amount of *t*-cinnamic acid, quercetin, chrysin and pinocembrin. Furthermore, it has been rich in ferulic acid and apigenin and therefore exhibits the highest DPPH (2,2-diphenylpicrylhydrazyl) radical scavenging activity. What is more, a highest amount of gallic and *p*-coumaric acid as well as apigenin probably has impact on the highest antioxidant activity of Polish propolis in assay with ABTS. Our study shows that propolis antioxidant activity is connected with that natural product origin, as well as its polyphenol content. The comparison between the antioxidant activity of propolis and well- known antioxidant such as ascorbic acid and BHT demonstrated that all samples of propolis exhibit powerful antioxidant activity. The obtained results pointed out that Polish propolis is similar to that from different provinces of Romania, while Turkish and Uruguayan ones are completely different, which seems to suggest that the mentioned differences originate from the diverse plants sources. The antioxidant activity was evidenced by in vitro methods, therefore these are preliminary studies that need later in vivo testing.

## Figures and Tables

**Table 1:** Total phenolic content and flavonoids in propolis extracts [mg/g dry extract].

Propolis Extracts Samples	Total Phenolic Content	Flavonoids Content
POL	123.00 ± 1.17	33.847 ± 0.83
TUR	135.982 ± 2.15	60.427 ± 0.45
URU	85.328 ± 0.37	48.443 ± 0.64
RO 1	129.65 ± 0.91	7.728 ± 1.15
RO 2	155.279 ± 0.25	10.493 ± 0.79
RO 3	128.444 ± 0.45	25.089 ± 1.07
RO 4	126.635 ± 0.81	23.399 ± 0.14
RO 5	123.922 ± 0.42	12.337 ± 0.18

POL-Poland; TUR-Turkey; URU-Uruguay; RO1–5—Romania. Values represent the average of three independent replications ±SD. The measurement has been performed in triplicates.

**Table 2:** Phenolic compounds detected in propolis extracts [µg/g dry weight, *n* = 3, ±SD, *p*-level of statistical significance] by HPLC analysis.

Phenolic	Propolis Extracts Samples								
	POL	TUR	URU	RO 1	RO 2	RO 3	RO 4	RO 5	*p*
Gallic acid	77.786 ± 1.830	11.199 ± 0.186	9.506 ± 0.332	30.240 ± 0.681	38.102 ± 1.022	38.777 ± 0.967	23.634 ± 0.365	30.420 ± 0.170	0.0023
Caffeic acid	420.345 ± 12.601	547.929 ± 15.028	89.611 ± 1.763	554.146 ± 19.222	778.371 ± 14.934	405.282 ± 10.080	453.133 ± 11.455	176.769 ± 4.781	0.0022
*p*-Coumaric acid	3452.608 ± 94.573	455.273 ± 16.510	277.124 ± 9.211	2860.897 ± 86.887	3547.561 ± 103.443	1823.821 ± 57.168	1835.579 ± 55.423	1530.515 ± 38.618	0.0023
Ferulic acid	314.906 ± 9.271	578.586 ± 13.088	119.007 ± 2.413	773.992 ± 19.886	1114.551 ± 44.281	545.946 ± 10.094	726.721 ± 30.413	157.101 ± 5.350	0.0020
Luteolin	-	10.547 ± 0.364	-	-	-	-	-	-	-
Quercetin	91.708 ± 2.150	138.984 ± 1.434	-	-	-	84.408 ± 3.513	56.158 ± 1.460	33.916 ± 0.652	-
*t-*Cinnamic acid	69.213 ± 2.563	235.758 ± 5.196	63.205 ± 1.254	48.953 ± 1.983	60.553 ± 1.154	67.230 ± 2.351	55.098 ± 0.373	23.354 ± 0.841	0.0021
Apigenin	219.721 ± 8.274	216.884 ± 7.032	124.953 ± 4.610	-	-	-	-	-	-
Hesperidin	-	69.263 ± 2.166	138.938 ± 3.397	-	-	-	-	-	-
Rhamnetin	-	199.954 ± 0.884	-	-	-	-	-	-	-
Chrysin	1902.704 ± 81.377	2817.433 ± 66.422	2020.016 ± 37.026	553.239 ± 22.673	993.915 ± 10.304	1259.567 ± 57.088	1114.312 ± 18.400	457.006 ± 14.201	0.0200
Pinocembrin	8.865 ± 0.276	2816.289 ± 95.501	1726.058 ± 73.542	125.600 ± 1.984	347.389 ± 10.513	916.312 ± 29.144	413.211 ± 6.774	223.297 ± 4.520	0.0190
Caffeic acid phenethyl ester (CAPE)	351.694 ± 8.555	1118.623 ± 35.908	344.625 ± 9.201	283.371 ± 11.156	348.457 ± 11.404	419.588 ± 7.958	435.229 ± 7.830	122.557 ± 2.161	0.0300

**Table 3:** Names and origin of propolis samples.

Name of Sample	Country	Region of Collect
POL	Poland	Beskid Mountains
TUR	Turkey	Anatolia
URU	Uruguay	Riviera department
RO 1	Romania	Transylvania, Sibiu County
RO 2	Romania	Transylvania, Sibiu County
RO 3	Romania	Transylvania, Sălaj County
RO 4	Romania	Transylvania, Cluj County
RO 5	Romania	Transylvania, Cluj County

## Data Availability

Not applicable.
